# Chimeric Antigen Receptor T Cell Exhaustion during Treatment for Hematological Malignancies

**DOI:** 10.1155/2020/8765028

**Published:** 2020-10-23

**Authors:** Chunyi Shen, Zhen Zhang, Yi Zhang

**Affiliations:** ^1^Biotherapy Center, The First Affiliated Hospital, Zhengzhou University, Zhengzhou 450052, China; ^2^Cancer Center, The First Affiliated Hospital, Zhengzhou University, Zhengzhou 450052, China; ^3^School of Life Sciences, Zhengzhou University, Zhengzhou 450052, China; ^4^Henan Key Laboratory for Tumor Immunology and Biotherapy, Zhengzhou 450052, China

## Abstract

Immunotherapy, especially based on chimeric antigen receptor (CAR) T cells, has achieved prominent success in the treatment of hematological malignancies. However, approximately 30-50% of patients will have disease relapse following remission after receiving CD19-targeting CAR-T cells, with failure of maintaining a long-term effect. Mechanisms underlying CAR-T therapy inefficiency consist of loss or modulation of target antigen and CAR-T cell poor persistence which mostly results from T cell exhaustion. The unique features and restoration strategies of exhausted T cells (Tex) have been well described in solid tumors. However, the overview associated with CAR-T cell exhaustion is relatively rare in hematological malignancies. In this review, we summarize the characteristics, cellular, and molecular mechanisms of Tex cells as well as approaches to reverse CAR-T cell exhaustion in hematological malignancies, providing novel strategies for immunotherapies.

## 1. Introduction

Based on the safety and effectiveness in clinical treatment, a CD19-targeting CAR-T cell therapy for treating relapsed or refractory B cell acute lymphoblastic leukemia (ALL) in both children and young adults has been approved by the U.S. Food and Drug Administration (FDA) in 2017 [1, 2]. This landmark development of CAR-T therapy for B cell malignancies benefitted from the phase 2 global ELIANA trial involved in 75 patients with refractory ALL. Notably, the overall remission rate in patients who received CAR-T cell infusion reached to 81%, with 59% 12-month relapse-free survival (RFS) and 76% overall survival (OS), respectively [3, 4]. Furthermore, in one of our clinical trials to investigate the safety and efficacy of CD19 CAR-T cell therapy in relapsed and refractory B cell lymphoma, the complete remission was observed in 6/14 patients at 3 months with 77% overall response rate [5]. Subsequently, various clinical trials expanding CAR-T indications to other hematological malignancies were carried out. However, disease relapses following CAR-T therapy becomes a severe problem limiting clinical curative effect which cannot be ignored. On the one hand, antigen-positive or negative relapses occur in patients which leads to resistance to CAR-T cell therapy [6, 7]. On the other hand, poor persistence and restricted function resulted from T cell exhaustion is also a common cause of relapse [8]. In this review, we discuss the characteristics of exhausted CAR-T cells in hematological malignancies, as well as the strategies to restore the function and prolong the survival of exhausted CAR-T cells.

## 2. Molecular and Functional Characteristics of CAR-T Cell Exhaustion

T cell exhaustion was firstly described in mice during lymphocytic choriomeningitis virus (LCMV) infection [9, 10]. Subsequently, similar findings were defined in human with chronic viral infection, as well as in cancers [11, 12]. Tex cells were characterized as a distinct population with loss of proliferation potential and effector function, multiple immune inhibitory receptors upregulation [13]. These features are used together for Tex cells definition.

### 2.1. Loss of Effector Function

It is clear that Tex cells are always lack of additional proliferation ability upon restimulation *in vivo*. Riches and his colleagues also found that T cells from chronic lymphocytic leukemia (CLL) patients displayed features of exhaustion with failure in proliferation and cytotoxicity [14]. In addition, T cells from acute myeloid leukemia (AML) patients exhibit loss of proliferation potential when cultured with allogeneic AML cells [15]. In a comprehensive analysis among the 41 patients with CLL who received CD19-directed CAR-T cell therapy, T cells from patients with nonresponding (NR) showed an exhaustion signature with limited proliferation. Furthermore, tumor-bearing mice with complete remission (CR) patients-derived CAR-T cells infusion exhibited stronger ability in reduction of tumor growth than CAR-T cells from NR patients [16]. Indeed, the activation and expansion of CAR-T cells after infusion are essential for remission in clinical.

### 2.2. Coexpression of Multiple Inhibitory Receptors

A major hallmark of Tex cells is high expression of inhibitory receptors including program cell death protein 1 (PD-1), cytotoxic T lymphocyte-associated protein 4 (CTLA-4), T cell immunoglobulin and mucin-domain containing-3 (Tim-3), lymphocyte activation gene-3 (Lag-3), and T cell immunoglobulin and ITIM domain (TIGIT) [17]. Several clinical studies and lots of preclinical research have described the exhaustion-related marker expression on CAR-T cells. In a chronic myeloid leukemia (CML) murine model, CML-specific T cells underwent exhaustion resulting from a PD-1 expression [18]. Furthermore, in a clinical trial involved in 43 pediatric and young adult subjects who received CD19 CAR-T cells, the NR group showed higher frequencies of Tim-3^+^ and Lag3^+^ CAR-T cells [19]. These findings indicated that inhibitory receptors restrict CAR-T cells activity and promote exhaustion.

## 3. Development of CAR-T Cell Exhaustion

**Figure 1:**
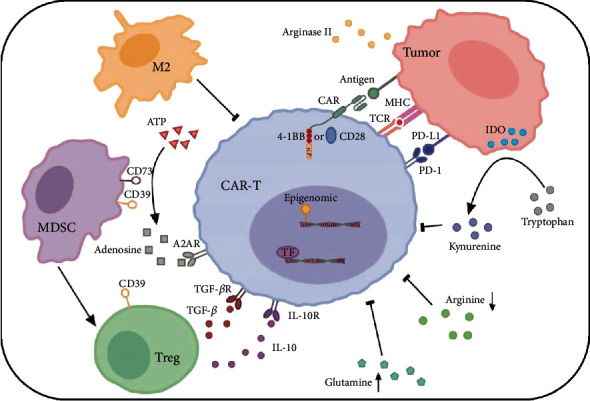
The factors involved in the development of CAR-T cell exhaustion. Persistent antigen from tumor cells interaction with TCR and CAR leads to hyperactivation, which drives expression of inhibitory receptors such as PD-1. Different costimulatory domains (such as CD28 and 4-1BB), epigenetic, and transcriptional profile modification are also involved in the process of CAR-T cell exhaustion. Immunoregulatory cells contribute to a tumor-supportive environment by producing suppressive cytokines such as IL-10 and TGF-*β* from Tregs that inhibit CAR-T cell activity and proliferation. Alteration of metabolic environment, including increase of adenosine by CD39 and CD73 in MDSCs, accumulation of kynurenine by IDO from tumor, limitation of arginine, and high level of glutamine, results in tumor cell survival and CAR-T cell dysfunction.

The factors driving T cell exhaustion seem to be complicated as a result of suppressive tumor microenvironment created by solid tumors. Similar phenomena appear to extend in several types of hematological malignancies including CLL, AML, and diffuse large B cell lymphoma (DLBCL) [20, 21]. The general pathways involved in the development of T cell exhaustion consist of persisting antigen stimulation, costimulatory domain of CAR structure, negative regulation by inhibitory receptors, immune suppressive factors, and immunoregulatory cells. However, additional factors such as transcriptional factors, metabolism, and epigenetic modification also play roles in CAR-T cell exhaustion development (see Figure 1).

### 3.1. Inhibitory Receptors in Tex Cells

Sustained expression of multiple inhibitory receptors is a key characteristic of Tex. It was established that tumor cells can escape through immune checkpoint pathways including CTLA-4 and PD-1 in hematological malignancies [21]. The PD-1 expression in CD19 CAR-T cells has already been described in clinical trials [22]. PD-1 limits CAR-T cell function when engagement with its ligand programmed death-ligand 1 (PD-L1) [23, 24]. Aberrant PD-L1 expression is not only observed in solid tumors but also detected in hematological malignancies including DLBCL, CLL, and AML [18, 25, 26], which creates opportunities for engagement of PD-1 and PD-L1. The PD-1 expression can be regulated by transcriptional factors (TF) and epigenetic modification. There is an additional enhancer in Tex cells which promotes the PD-1 expression [27, 28]. Upregulation of T-bet is consistent with PD-1^int^Eomes^lo^ Tex formation, while Eomes is elevated in the more terminal Tex subset [29]. NFAT is a transcription factor family with a clear effect in T cell exhaustion, which binds to PD-1 promoter to induce inhibitory receptor expression [30]. Recently, it has been demonstrated that relative deficiency in c-Jun–c-Fos AP-1 heterodimers related to T cell exhaustion. Overexpression c-Jun in CAR-T cells reduced the PD-1 expression, restored *in vitro* effector function, reversed exhaustion, and improved *in vivo* cytotoxicity against tumor cells in different leukemia models [31, 32]. Furthermore, in a murine model with AML, B7-1, the specific ligand of CTLA-4, is increased in tumor cells, which mediated the resistance to immune response and poor survival [33]. In addition, the CAR-T cells from murine model involved in AML showed a significant upregulation of the Tim-3 expression when disease relapsed after CAR-T cell infusion [26, 34]. Meanwhile, it was reported that Galectin 9, which was the ligand of Tim-3, was increased in AML blasts for immune surveillance escape [35, 36]. Therefore, based on the expression of inhibitory receptors in hematological malignancies, the application of immune checkpoint blockade has a potential for combination therapies with CAR-T cells.

### 3.2. Metabolic Challenges

T cells experience metabolic reprograming in response to stimulation from surroundings during differentiation. When naïve T cells (Tn) are activated, metabolic program transition from mitochondrial dependent oxidative phosphorylation to glycolysis for effector T cells (Teff) [37–39]. Subsequently, the formation of memory T cells (Tm) returns the metabolism state to oxidative phosphorylation and fatty acid oxidation [40–42]. However, Tex cells display aberrant metabolic reprograming. The upregulation gene signature in CD19 CAR-T cells from NR CLL patients was enriched in glycolysis [16]. In contrast, a research described that B cell leukemia decreased T cell glucose metabolism through inhibition of AKT/mTORC1 signaling [43]. And the impaired glucose uptake was observed in CLL-derived T cells. In addition, CAR-T cells from the CR CLL group presented significantly higher mitochondrial mass compared with NR patients [44]. Furthermore, tumor cells and immunosuppressive cells foster a hostile metabolic environment which is not suitable for T cell survival. AML blasts create a microenvironment with low arginine level through releasing arginase II. This severe environment induces M2 macrophage polarization and T cell dysfunction [15]. However, targeting the arginine metabolism reverses the anti-CD33 CAR-T cell antitumor capacity [45]. Adenosine enriched in leukemia microenvironment is regulated by ectonucleotidases CD39 as well as CD73, which results in T cell suppression through interacting with adenosine 2A receptors (A2AR) [36]. In addition, there were reports indicating that limitation of glutamine enhances antitumor function [46]. These findings reveal that cellular metabolism homeostasis has a strong link with clinical outcome, suggesting that metabolic alterations can be treated as targets for immunotherapies.

### 3.3. Antigen Persistence and Costimulatory Signaling

Persistent antigen exposure is a key feature that contributes to T cell exhaustion in majority cases of chronic virus infection and cancers [47]. Although CAR-T cell effector function is independent on major histocompatibility complex-restricted specificity, it has been reported that CAR-T cells can also be driven by endogenous T cell receptor (TCR) signaling. In a model of CD19 CAR-T cell therapy for pre-B cell ALL, CAR-T cells underwent exhaustion at the presence of TCR antigen stimulation [48]. However, in spite of persisting antigen stimulation, tonic CAR signaling with antigen independence can also trigger early exhaustion of CAR-T cells which impairs antitumor efficacy. This results from the costimulatory domain, including CD28 and 4-1BB, exhibiting different impact upon T cell exhaustion, respectively [49]. Various clinical trials have involved in the efficacy of CAR-T cells with CD28 (28*ζ*) and 4-1BB (BB*ζ*) costimulatory domains. Although patients with DLBCL showing a CR of 57% at one month after 28*ζ* CAR-T treatment, a high recurrence rate was observed during follow-up [50], while patients treated with BB*ζ* CAR-T cells reached over 40% completed remission at six months [51]. 4-1BB domain promoted the formation of memory CAR-T cells *in vivo*, improving 86% of DLBCL patients to achieve sustained remission at a median follow-up of 28.6 months [51]. Furthermore, BB*ζ* CAR-T cells targeting CD19 continued to be detectable over 4 years after infusion in some patients with CLL, while 28*ζ* CAR-T cells have a survival time of 12 months *in vivo* [50, 52]. In addition, 4-1BB can ameliorate T cell exhaustion and prolong CAR-T cell survival [49]. Not only CD28 and 4-1BB but other costimulatory domains including OX40, CD27, and ICOS also regulate persistence and effector function of CAR-T cells [53]. Compared with CD28 domain, CAR-T cells harbored CD27 domain show elevated persistence *in vivo* while ICOS promotes Th17/Th1 cell differentiation [54, 55]. Indeed, incorporation of two costimulatory domains can enhance the CAR-T cell activity. Combination of CD28 and 4-1BB domains in CD19 CAR-T cells showed more robust expansion and longer persistence than CD28 only in patients with lymphoma [56]. Similarly, the ICOS-4-1BB-based CAR-T cells also have superior effector function against tumor cells [57]. In CD30-targeting CAR-T cells, CD28 and OX40 costimulatory combination promotes antitumor efficacy and improves persistence and proliferation *in vivo* against CD30^+^ lymphoma [58]. Therefore, selection of costimulatory domain also impacts the efficacy of CAR-T cell therapy, which is an approach for prevent CAR-T cells from exhaustion.

### 3.4. Tumor Microenvironment Factors

Tumor microenvironment contains various kinds of immune regulatory factors shaping T cell exhaustion. CLL, due to its suppressive microenvironment with imbalanced immune system, shows low response towards CAR-T cell therapy [26]. The expansion of myeloid-derived suppressor cells (MDSCs) was observed in CLL patient peripheral blood. This subset of MDSCs promoted regulatory T cell (Treg) and secreted high level of indoleamine 2,3-dioxygenase (IDO) which limited T cell proliferation and cytotoxicity through catabolizing tryptophan degradation [59]. A prevalent population of Treg emerging in AML microenvironment interferes T cell function [60]. The IL-10 and TGF-*β* released by Treg lead to CAR-T cell dysfunction [61]. When clearance of Tregs in AML mouse model, the antitumor efficacy of infused cytotoxic T cells was improved, suggesting its immunosuppressive capacity against T cells [62]. The M2 macrophages contribute to creating a tumor-supportive microenvironment. M2 macrophages from leukemia mouse model better improved leukemia cell lines expansion *in vitro* than macrophages from normal mice [63]. It has been demonstrated that M2 macrophages in B cell lymphoma express PD-L1, which indicates that they can directly inhibit T cell function through PD-1/PD-L1 interaction [64]. In addition, Tregs are recruited into tumor microenvironment through CCL22 secreted by M2 macrophages [20]. In a clinical trial enrolled in 10 refractory B cell lymphoma patients receiving CD19 CAR-T cells, infiltration of tumor-associated macrophages related negatively with remission status, highlighting macrophages reducing the CAR-T cell efficacy [65]. Furthermore, tumor-associated macrophages have been revealed playing a role in mediating cytokine release syndrome [66], indicating that macrophages can act as an indicator for clinical response. In addition to antigen stimulation and inhibitory receptors, it seems that diversity of immune suppressive factors also plays key roles in shaping T cell exhaustion, which must be taken into consideration when prevent CAR-T cells from exhaustion.

## 4. Emerging Strategies to Reverse CAR-T Cell Exhaustion

Although the treatments against hematologic malignancies such as chemotherapy, targeted therapies including rituximab, and stem cell transplantation prolong survival, patients still have a poor prognosis. CAR-T cell therapy shows a remarkable clinical outcome [67]. However, the problems including CAR-T cell expansion limitation, short-term remission, and T cell exhaustion restrict the therapeutic effect [6]. The applications for reinvigorating Tex cells are based on the development of exhaustion. Immune checkpoint regulation, T cell differentiation, and engineering provide targets for T cell modification.

### 4.1. Combination Therapy of Immune Checkpoint Blockade and CAR-T Cells

There have been several open clinical trials exploring the effect of immune checkpoint blockade combined with CAR-T cell therapy. A patient with refractory DLBCL after receiving CD19 CAR-T cell therapy was treated with PD-1 blocking antibody, resulting in a significant CAR-T cell expansion, strong antitumor response, and decreased tumor burden [68]. Another patient with follicular lymphoma did not benefit from CAR-T cells monotherapy. After receiving a low-dose PD-1 blockade, patient achieved remission lasting for more than 10 months without severe side effect [69], suggesting that precise dose of PD-1 blockade should be taken into account during clinical use. The combination of CD19 CAR-T therapy with PD-1 blockade in 14 ALL patients who were failure in CAR-T therapy showed better response with persistence of CAR-T cells [70]. The addition of PD-1 blockade to CD19 CAR-T therapy in 6 pediatric B-ALL patients augmented response to CAR-T cells and half of the patients had improved clinical outcomes [26]. Despite of PD-1/PD-L1 pathway, CTLA-4/B7-1 interaction in murine leukemia model inhibited T cell response. Blocking CTLA-4 enhanced antileukemia responses and prolonged survival in mice [70]. In patients with relapsed AML after autologous stem cell transplant, CTLA-4 inhibitor ipilimumab showed effective in partial patients [71]. The Tim-3 expression was upregulated in CAR-T cells from relapsed AML murine model after CAR-T cell infusion. Then, adding Tim-3 blockade to CAR-T cell therapy led to enhanced antitumor efficacy [34]. The increased Lag3 expression was detected in T cells from CLL tumor microenvironment, and blocking Lag3 improved T cell activation [72]. These emerging evidences indicated that combining PD-1 blockade with CAR-T cells could enhance benefit in exhausted T cell reinvigoration, even though other inhibitors are not used as widely as PD-1 blockade. However, given that these immune checkpoint blockades showed effectively in preclinical studies, they are expected to be combined with CAR-T cell therapy or synergize with anti-PD-1 blocking for antitumor treatment.

### 4.2. Gene Modification for Overcoming CAR-T Cell Exhaustion

The exhausted T cells represent a unique subset with aberrant receptors expression and distinct pathways activation that are different from Tm or Teff cells. These factors resulting in CAR-T cell exhaustion can be modified by gene engineering technology. To overcome the limitation of PD-1 pathway, Li et al. engineered anti-PD-1 antibody secreting CAR-T cells, which enhanced the antitumor capacity [73]. Furthermore, depletion of PD-1 by CRISPR/Cas9 improved the effector function against tumor in anti-CD19 CAR-T therapy [74, 75]. Schlenker et al. have changed the chimeric receptors as PD-1 extracellular domain with the CD28 signaling domain. This design can enhance the cell proliferation and antitumor response [76]. The transcriptional factors NR4A family and high-mobility group-protein TOX are related with T cell exhaustion. NR4A activation is correlated with inhibitory receptor expression whereas TOX drives epigenetic programming of Tex cells. Recent studies showed that NR4A-knockout CAR-T cells had low expression of inhibitory receptors and reduced tumor growth [77]. The effector function of CAR-T cells was improved after TOX depletion [78]. The modification of costimulatory molecules also has an impact on CAR-T cell activity. In addition to the known costimulatory molecules, other novel modified costimulatory molecules are explored. Toll-like receptors (TLRs) can serve as costimulatory molecules to augment T cell cytokines secretion. CD19 CAR-T cells with costimulatory signaling domains containing CD28 and TLR2 exhibited enhanced effector function and expansion capacity [79, 80]. Cytokine engagement is also involved in T cell activation. Hence, Kagoya et al. engineered a CD19 CAR construct harboring a domain of IL-2R*β* and a STAT3-binding YXXQ motif excepted of CD3z and CD28 domains, which strengthened the effector function and persistence of CAR-T cells [81]. IL-15 is an effective factor that contributes to T cell survival and Tm differentiation. Incorporation of costimulatory molecules CD28 and IL-15R*α* showed lasting killing and expansion activities compared with other combinations [82]. Gene engineering CAR-T cells overcome partial mediators contributing to exhaustion which is a promising strategy for clinical use. Since multiple factors are involved in driving T cell exhaustion, it is necessary to find critical molecules that play a major role in regulating exhaustion, which act as important targets for gene engineering.

### 4.3. CAR-Tscm Generation

Tm cells show advantages in immunotherapy due to its early differentiation stage, long survival ability, and strong antitumor effect [83]. T memory stem cell (Tscm), developing from Tn cells, has a potential to differentiate into Tm and Teff [84]. This subset of cells shows stronger antitumor effect than Tn cells while displays more potent proliferation and self-renewal capacity than Tm and Teff cells [85]. Thus, expansion *in vitro* and genetic engineering of Tscm cells are a strategy for potent CAR-T cells generation. Tscm can be induced by interleukin-7 (IL-7), IL-15 or IL-21, and the glycogen synthase-3*β* inhibitor TWS119 in culture system. Engineered CD19 CAR-T cells with Tscm exhibited improved metabolic fitness and induced robust, persisting antitumor response against ALL [86, 87]. Use of younger T cells provides a novel source for stronger CAR-T cell generation. By studying mechanisms of differentiation and induction of Tm cells, Tm cell culture condition is continuously optimized. However, the safety of different cocktails in culture system should take into consideration for clinical application.

### 4.4. Targeting Tumor Microenvironment

The tumor-supporting microenvironment has severe surroundings containing soluble factors and immunoregulatory cells which suppressive CAR-T cell function. The low arginine microenvironment limits CAR-T cell efficacy. CAR-T cells with low expression of the arginine resynthesis enzymes are susceptible to this microenvironment. CAR-T cells which are modified to express arginine resynthesis enzymes for regulating metabolic alteration show increased proliferation and enhanced clearance of leukemia *in vivo* [88]. Furthermore, the increase of MDSCs in AML patients inhibits T cell responses. T cells with CD33/CD3-bispecific BiTE antibody construct have been proved to eliminate CD33^+^ MDSCs while against AML blasts [89]. In addition, IDO derived from tumor cells and immunosuppressive cells interferes T cell activity. Combination CD19 CAR-T cells with IDO inhibitor (1-methyl-tryptophan) or fludarabine and cyclophosphamide improves antitumor efficacy of CAR-T cells in lymphoma [90]. Notably, monotherapy only targeting tumor cells cannot achieve durable disease control. Modifying tumor microenvironment is a promising approach for cancer treatment. Rational combinations of cellular therapy and tumor microenvironment modification are expected to be the next generation of tumor immunotherapy.

## 5. Conclusion

T cell exhaustion plays a critical role in immune evasion and dysfunction, resulting in a low clinical response and poor outcomes. Revealing the underlying mechanisms leading to exhaustion has provided novel therapeutic approaches for recovery of CAR-T cell cytotoxicity and persistence. The advance of Tex cells research benefits from the development of epigenetic analysis and transcriptomics sequencing technology. Actually, Tex cells exhibit unique epigenetic and distinct transcriptional landscape which are different from Teff and Tm. Thus, key molecules driving T cell exhaustion based on the epigenetic or transcriptional regulation are expected to become the targets for editing. Despite of modifying the CAR-T cells, targeting restricted factors to reverse the immune suppressive tumor microenvironment is also a promising strategy. Therefore, it is important to keep on dissecting molecular and cellular mechanisms resulting in the T cell exhaustion, reprogram the pathways that promoting T cell dysfunction, and further optimize CAR-T cell immunotherapy.

## Data Availability

The data supporting this review are from previously reported studies and datasets, which have been cited at relevant places within the text as references.

## Abbreviations

CAR-T: Chimeric antigen receptor T cell
Tex: Exhausted T cell
ALL: Acute lymphoblastic leukemia
CLL: Chronic lymphocytic leukemia
AML: Acute myeloid leukemia
DLBCL: Diffuse large B cell lymphoma
CML: Chronic myeloid leukemia
FDA: U.S. Food and Drug Administration
RFS: Relapse-free survival
OS: Overall survival
LCMV: Lymphocytic choriomeningitis virus
NR: Nonresponding
CR: Complete remission
PD-1: Programmed cell death 1
PD-L1: Programmed cell death ligand 1
CTLA-4: Cytotoxic T lymphocyte-associated protein 4
Tim-3: T cell immunoglobulin and mucin-domain containing-3
Lag-3: Lymphocyte activation gene-3
TIGIT: T cell immunoglobulin and ITIM domain
TF: Transcriptional factor
Tn: Naïve T cell
Teff: Effector T cell
Tm: Memory T cell
TCR: T cell receptor
MDSC: Myeloid-derived suppressor cell
Treg: Regulatory T cell
IDO: Indoleamine 2,3-dioxygenase
A2AR: Adenosine 2A receptor
Tscm: T memory stem cell.
